# Strategies for Transvenous Lead Extraction Procedures

**DOI:** 10.19102/icrm.2017.080502

**Published:** 2017-05-15

**Authors:** Laurence M. Epstein, Melanie Maytin

**Affiliations:** ^1^Department of Medicine, Division of Cardiovascular Medicine, Brigham and Women’s Hospital, Boston, MA

**Keywords:** Defibrillator, lead extraction, lead management, pacemaker

## Abstract

Transvenous lead extraction (TLE) has undergone an explosive evolution since its inception as a rudimentary skill with limited technology and therapeutic options. Early techniques involved simple manual traction that frequently proved ineffective for chronically implanted leads, and carried a significant risk of myocardial avulsion, tamponade, and death. The morbidity and mortality associated with these early extraction techniques limited their application to use only in life-threatening situations, such as infection and sepsis. The past four decades, however, have witnessed significant advances in lead extraction technology, resulting in more efficacious techniques and tools, providing the skilled extractor with a well-equipped armamentarium. With the development of the discipline, we have witnessed a growth in the community of TLE experts coincident with a marked decline in the incidence of procedure-related morbidity and mortality, with recent registries at high-volume centers reporting high success rates with exceedingly low complication rates. Future developments in lead extraction are likely to focus on new tools that will allow for us to provide comprehensive device management, develop alternative systems for extraction training, and focus on the design of new leads conceived to facilitate future extraction.

## Lead management

The expansion of indications for cardiac implantable electronic device (CIED) therapy and an aging population have resulted in an exponential increase in CIED use. However, with this, observed complications have also increased in a disproportionate manner^1,2^ because of greater CIED utilization, more frequent device system revisions for complications,^3–5^ system upgrade,^6–8^ lead malfunction,^9–13^ and longer patient life expectancies. This has mandated a paradigm shift towards premeditated lead management strategies—from implant to removal or replacement. In fact, it is estimated that the demand for transvenous lead extraction (TLE) has reached an annual rate of nearly 24,000 patients worldwide (B. Safyan, personal communication, Spectranetics).

### TLE indications

As advances in extraction techniques have made lead removal both safer and more successful, TLE indications have expanded to include more clinical situations.^14^ The 2009 HRS Expert Consensus Statement on Transvenous Lead Extraction extended class I indications to include patients with CIED pocket infection, occult Gram-positive infection, and functional leads that, owing to design or failure, may pose an immediate threat if left in place **(Table 1)**. The class II indications for TLE were further divided into class IIa (reasonable necessity to perform the procedure) and class IIb (may consider performing the procedure) indications. Class IIb indications for TLE include CIED patients with superfluous functional or non-functional leads, those with functional leads that pose a risk of device interference, and/or those whose devices (owing to design or failure) pose a potential future risk of adverse events. It is important to note that the Consensus document is in the process of being revised, although major changes in indications for TLE are not anticipated to occur.

**Figure 1:**
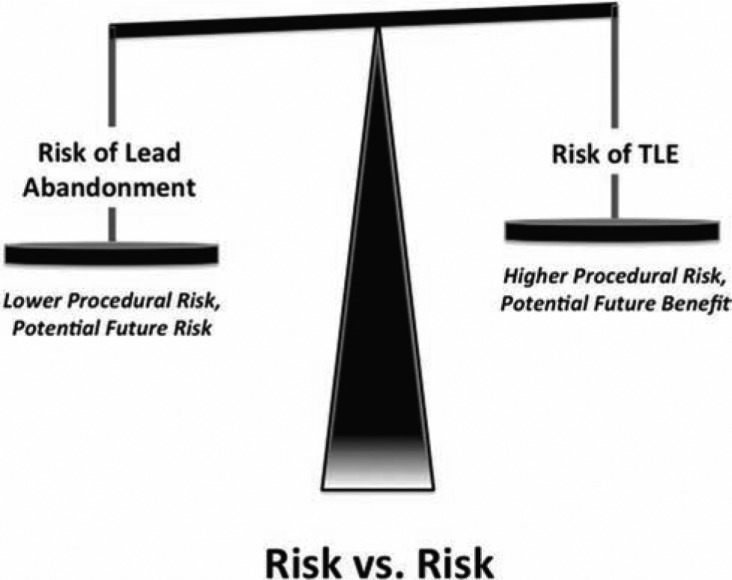
Risk versus risk. The decision regarding lead extraction or abandonment requires comparison of the current risks of lead extraction with the future risks of both lead abandonment and potential lead extraction.

In assessing an individual’s indication for TLE, comparing the risks of extraction with the risks of lead abandonment is mandated **(Figure 1)**. The consideration of patient and lead characteristics and, importantly, operator experience, must be factored into the TLE risk assessment. The risk assessment evaluation must include specific attention to the number of leads, the implant duration, the use of defibrillator versus pacing electrodes, patient age, and any comorbidities.

Decisions regarding lead extraction must be made on a case-by-case basis, integrating various patient and lead characteristics and operator-related variables. Lead extraction with the potential for significant morbidity and mortality may not be warranted in patients with a poor prognosis or in those in which the risks of intervention clearly outweigh the risks of lead abandonment. Additionally, operators who are inexperienced in the procedure should not perform lead extraction, nor should those who lack the necessary tools available to attain complete success or those who are in a practice setting not adequately prepared and committed to the complete and safe performance of the procedure perform such a task.^14^

### Pre-procedure preparation

Transvenous lead extraction, like any surgical procedure, requires a team-oriented approach that anticipates and plans for all potential scenarios. The required personnel of the extraction team include the following, at minimum: the physician who will perform the extraction, a cardiothoracic surgeon (if this person is not already the primary operator), an individual to provide anesthesia support, an X-ray technician or other person to operate the fluoroscopy, and both scrubbed and non-scrubbed assistants. Although complications will occur, the single most important factor in preventing a major complication from leading to a death is the time to intervention. Thus, the procedure location, e.g. the operating room or catheterization or electrophysiology laboratory, is less important than the immediate availability of cardiothoracic surgical intervention. This contingency mandates that a surgeon proficient in managing the potential complication be on-site during the extraction procedure, and that the equipment necessary for cardiopulmonary bypass be readily available. Additional emergency equipment is required to be present in the room or at an immediate availability, including transthoracic and/or transesophageal echocardiography, a pericardiocentesis tray, vacuum containers for chest tube drainage, temporary pacing equipment, an anesthesia cart for general anesthesia, and vasopressors and other emergency medications. We have fashioned a mobile “extraction cart” **(Table 2)** that contains all of the aforementioned emergency equipment, in addition to extraction tools (i.e. locking stylets, powered and non-powered sheaths, femoral workstations, and extraction snares) and CIED implant tools (e.g. stylets, wrenches, fixation tools, introducer sheaths, intravenous contrast materials, and repair kits).

**Figure 2:**
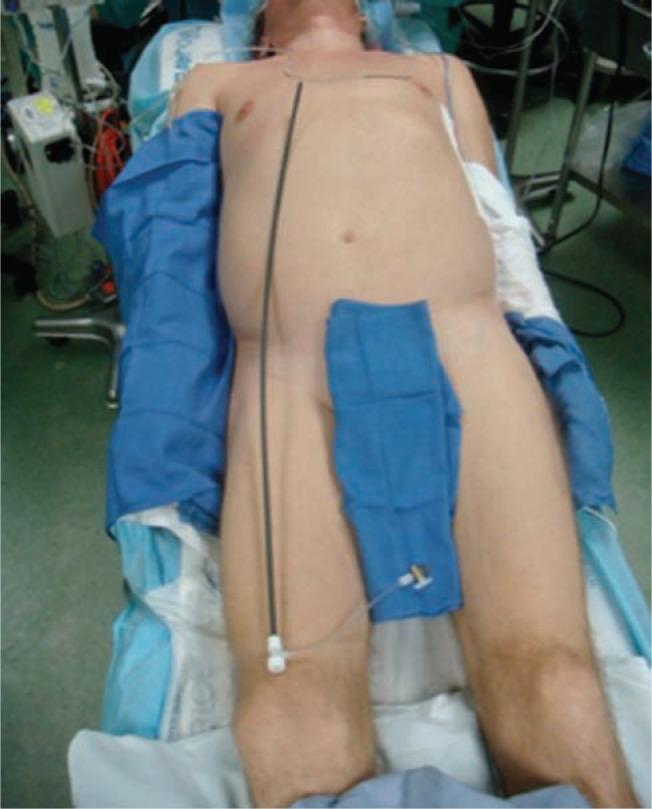
Patient preparation. We prepare our patients with a chlorhexidine solution and drape so as to allow for access for contralateral implant or for emergent pericardiocentesis, thoracentesis, thoracotomy, sternotomy, or cardiopulmonary bypass.

Recognizing the potential need for emergent surgical intervention, our patients are prepared for the procedure in such a way so as to eliminate any delays. We prepare our patients with a chlorhexidine solution and drape, so as to allow for access for contralateral implantation or emergent pericardiocentesis, thoracentesis, thoracotomy, sternotomy, or cardiopulmonary bypass **(Figure 2)**. It is our practice that patients are to have bilateral peripheral venous access with large bore catheters, femoral venous access, invasive hemodynamic monitoring with a radial arterial line, general endotracheal anesthesia, and four units of packed red blood cells readily available. In cases expected to be more complex or characterized by a higher risk of complications, we will advance an Amplatz Super Stiff™ guidewire (Boston Scientific, Marlborough, MA, USA) to the level of the right internal jugular or either innominate vein via the femoral vein.

Prior to the scheduling of the procedure, many patient-specific factors must be considered. In addition to the usual preoperative considerations, one must also be sure of what hardware is actually implanted. Some leads require certain tools, such as stylets, to retract an active fixation screw. Registered device/lead information can be inaccurate, and abandoned leads may not be properly identified. Thus, we recommend a chest X-ray be performed in all patients prior to surgery to identify what hardware is present and its anatomic location(s). If there are any concerns that a lead may no longer be intravascular or cardiac, the use of further imaging modalities such as computed tomography (CT) is warranted. Additionally, the following should be considered: (1) Does the patient have a history of prior cardiac surgery? This reduces the risk of perforation but increases the difficulty of surgical intervention if required. (2) Are you going to reimplant at the time of extraction, and, if so, what is the plan for vascular access? If the implant vein is occluded, removing the lead with any traction may cause you to lose access. (3) Will the patient require temporary pacing for the procedure, or for some time afterward, in case of infection?

### TLE tools

There is no one “right” tool for lead extraction. Each patient presents a unique clinical environment; thus, having all of the available tools is important to success. While many extractors have individual preferences for a given situation, all experienced extractors agree that having a full “quiver” of tools is necessary in order to achieve the highest possible success rate safely.

#### Locking stylets

**Figure 3:**
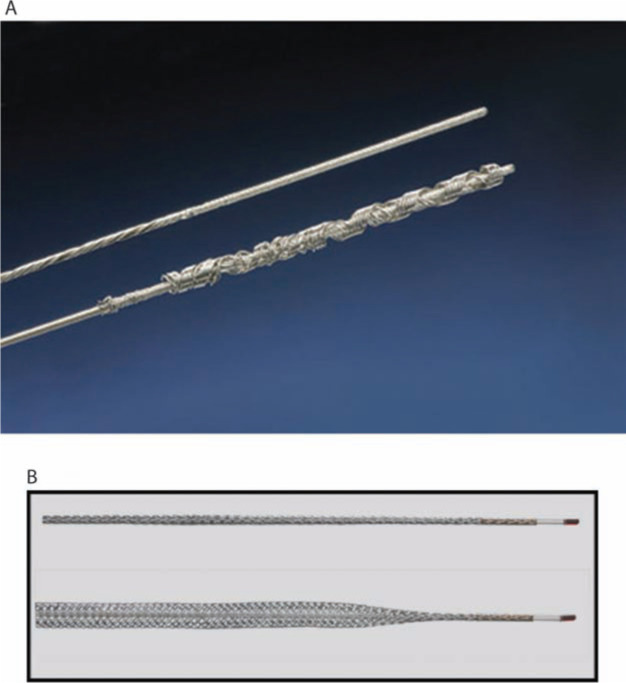
Locking stylets for transvenous lead extraction. **A:** The Liberator^®^ Locking Stylet (Cook Medical, Bloomington, IN, USA) fits leads with lumen diameters of 0.016 to 0.032 inches. An undeployed Liberator^®^ locking stylet is shown above a deployed Liberator^®^ locking stylet. When deployed, the wound spring at the end of the stylet opens up, locking into place. Courtesy of Cook Medical, Inc. **B:** The Lead Locking Device (LLD^®^) EZ (Spectranetics, Colorado Springs, CO, USA) has a radiopaque tip and accommodates inner coil diameters of 0.015 to 0.026 inches (undeployed stylet, top). In contrast to the Liberator^®^ locking stylet, the LLD^®^ locking stylet has a braided mesh over the entire length of a solid lead that expands when deployed (bottom). Courtesy of The Spectranetics Corporation.

The ability to successfully extract a lead with traction is directly dependent upon the lead construction and its tensile strength.^15^ Locking stylets were developed to reinforce the lead, transmit the extraction force to the tip of the lead, reduce the risk of lead disruption, and increase the likelihood of complete lead removal.^16–18^ Several types of locking stylets have been designed. While the original locking stylets had to be sized to the luminal diameter of the conductor coil, the most commonly utilized locking stylets today are designed to accommodate a range of conductor coil diameters. The Liberator^®^ (Cook Medical, Bloomington, IN, USA) and Lead Locking Device (LLD^®^) EZ (Spectranetics, Colorado Springs, CO, USA) stylets offer similar support, but differ in their locking mechanism design. The locking mechanism of the Liberator^®^ (Cook Medical, Bloomington, IN, USA) is located at the distal tip of the stylet, providing focal traction at the tip of the lead, whereas the LLD^®^ EZ stylet grabs the lead in multiple areas and exerts force along the length of the lead **(Figures 3A and 3B)**.

**Figure 4:**
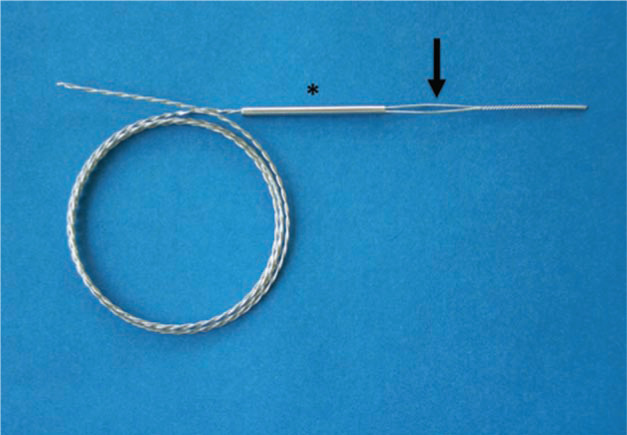
The Bulldog™ Lead Extender (Cook Medical, Bloomington, IN, USA). This useful tool is meant for leads that cannot receive a locking stylet, owing to either extensive damage or a solid-core design. The exposed end of the lead is passed through the loop of the Bulldog™ (arrow), and the metal sleeve (asterisk) is advanced over the loop grasping the lead. Courtesy of Cook Medical, Inc.

**Figure 5:**
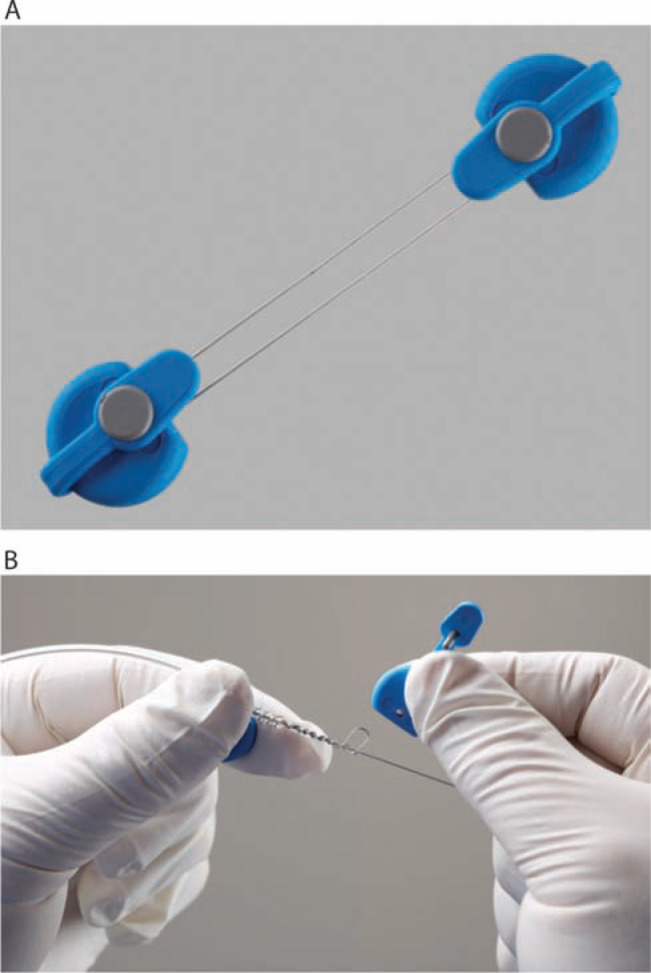
One-Tie^®^ Compression Coil (Cook Medical, Bloomington, IN, USA). **A:** Undeployed tool. **B:** The One-Tie^®^ Compression Coil (Cook Medical, Bloomington, IN, USA) is wound around the lead, compressing its components.

If a lead cannot receive a locking stylet (either because of extensive damage or because of a solid-core design), applying sufficient traction to it can prove challenging. The Bulldog™ Lead Extender (Cook Medical, Bloomington, IN, USA) is a tool that can be useful in such situations **(Figure 4)**. It consists of a wire with a threadable handle, through which the lead is passed and secured, thereby locking the insulation and conductor to the extender. The One-Tie^®^ Compression Coil (Cook Medical, Bloomington, IN, USA) is a useful tool for improving lead control, as it binds the proximal components of a lead together and to a locking stylet or the Bulldog™ Lead Extender (Cook Medical, Bloomington, IN, USA) **(Figures 5A and 5B)**.^19^

The advent of locking stylets has permitted safer and more successful transvenous lead extraction via the implant vein, stimulating the development of related techniques and technologies.

#### Telescoping sheaths

**Figure 6:**
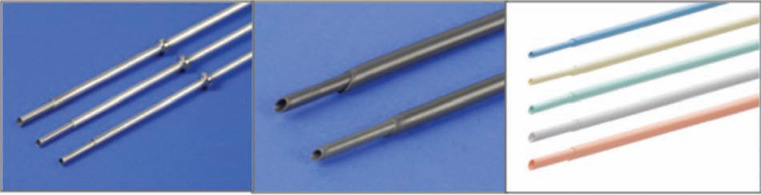
Telescoping non-powered countertraction sheaths. Telescoping sheaths are available in a range of sizes from 7F to 16F, and are made of different materials with varying properties, including **A:** stainless steel; **B:** Teflon™ (Chemours Co., Wilmington, DE, USA); and **C:** polypropylene. Courtesy of Cook Medical, Inc.

Telescoping sheaths are non-powered sheaths that are available in a range of sizes from 7F to 16F. They are made of different materials with varying properties, including stainless steel, Teflon™ (Chemours Co., Wilmington, DE, USA) and polypropylene **(Figures 6A, 6B and 6C).** As a material, Teflon™ (Chemours Co., Wilmington, DE, USA) is soft and flexible, but is unable to cut through dense scar tissue; in contrast, polypropylene is stiffer and better at disrupting encapsulating scars, but must be used with caution so as to avoid vascular injury. Stainless steel sheaths are employed only for disrupting dense and calcified fibrosis as the central venous circulation is entered. During typical use, the inner and outer sheath pair is advanced along the lead with alternating counterclockwise and clockwise motions in combination with the application of moderate pressure. The soft inner sheath is used as a guide, while the more rigid outer sheath serves to disrupt and dilate the encapsulating fibrous tissue. Sufficient traction is essential to ensure that the sheaths track the path of the lead and remain within the confines of the vasculature under fluoroscopic guidance. Using telescoping sheaths, TLE success rates via a superior (e.g. implant vein) approach range from 71% to 97%.^16,17,20,21^

#### Powered sheaths

**Figure 7:**
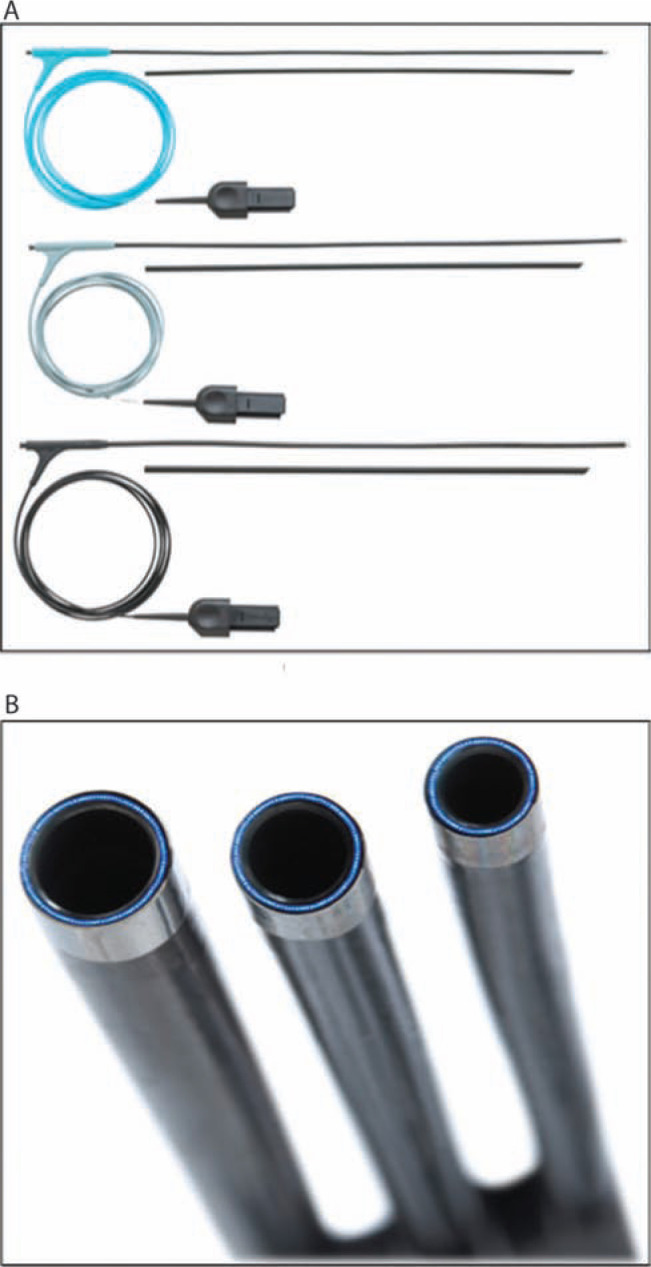
SLS^®^ II Excimer Laser Sheaths (Spectranetics, Colorado Springs, CO, USA). **A:** The Excimer laser sheath utilizes ultraviolet laser energy to vaporize tissue in contact with the tip of the sheath, where the optical fibers terminate. The sheath is available in a range of sizes (12F, 14F, and 16F), displayed from top to bottom. **B:** End-on view of the laser sheath showing the distal end, where the optical fibers terminate. Both images courtesy of The Spectranetics Corporation.

**Figure 8:**
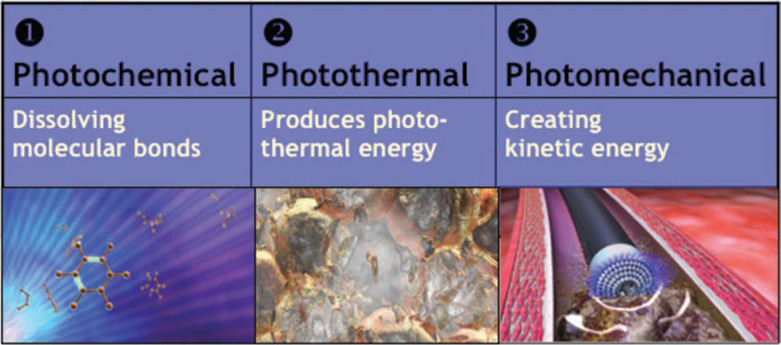
Mechanism of photoablation. The laser sheath applies circumferential pulses of energy at its distal end. The ultraviolet energy disrupts molecular bonds to a depth of 50 mm, causing cells to rupture and fibrotic tissue to dissolve, forming a vapor bubble. The vapor bubble expands and implodes, clearing debris from the distal end of the sheath. Courtesy of The Spectranetics Corporation.

Powered sheaths employ a source of energy to make the dissection of encapsulating fibrous tissue easier and more efficient. They enable the advancement of the sheath along the lead with reduced countertraction and counterpressure forces.^15,22^ One such powered sheath is the SLS^®^ II Excimer Laser Sheath (Spectranetics, Colorado Springs, CO, USA), allowing for the use of a “cool” pulsed ultraviolet laser at a wavelength of 308 nm during lead removal that is available in 12, 14, and 16F sizes **(Figures 7A and 7B)**. The laser sheath applies circumferential pulses of energy from its distal end. This energy dissolves tissue that comes into contact with the tip of the sheath by photochemical destruction of molecular bonds and photothermal ablation that vaporizes water and ruptures cells with resultant photomechanical creation of kinetic energy **(Figure 8)**.^23^ The sheath is advanced over the lead body, utilizing the standard techniques of counterpressure and countertraction, and laser energy is delivered when encapsulating fibrous tissue halts sheath advancement. Tissue in direct contact with the sheath tip is ablated to a depth of 50 mm until the distal electrode is reached; countertraction is still necessary to dislocate the lead tip. Compared with mechanical telescoping sheaths, laserassisted extraction resulted in more frequent complete lead removal, and shortened extraction times without an increase in procedural risk.^24–26^ The introduction of laser extraction changed the landscape of transvenous extraction, providing a highly effective and low-morbidity technique with broad applications.^24,26,27^ In 2012, a high-frequency laser sheath with twice as many pulses per second as the currently available laser sheath was released. The 80-Hz GlideLight™ laser sheath (Spectranetics, Colorado Springs, CO, USA) more efficiently advances through scar tissue and necessitates significantly less forward force on the sheath than its predecessor.^28–30^

The Perfecta^®^ Electrosurgical Dissection Sheath (Cook Medical, Bloomington, IN, USA) represents another type of powered sheath. The electrosurgical dissection sheath consists of an inner polytetrafluoroethylene (PTFE) sheath with bipolar tungsten electrodes exposed at the distal tip and an outer sheath for counterpressure and countertraction. Radiofrequency energy is delivered between the bipoles to dissect through fibrous binding sites, much like a surgical cautery tool, although the lead tip must be liberated with countertraction. In contrast to the SLS^®^ II Excimer Laser Sheath (Spectranetics, Colorado Springs, CO, USA), the Perfecta^®^ Electrosurgical Dissection Sheath (Cook Medical, Bloomington, IN, USA) permits localized application of radiofrequency energy with linear rather than circumferential dissection of the encapsulating fibrous tissue. The focused and steerable dissection plane offers the potential advantages of improved precision and diminished risk. The Perfecta^®^ Electrosurgical Dissection Sheath (Cook Medical, Bloomington, IN, USA) is also a cost-effective alternative to the SLS^®^ II Excimer Laser Sheath (Spectranetics, Colorado Springs, CO, USA) that does not compromise safety or efficacy.^31^

#### Hand-powered sheaths

**Figure 9:**
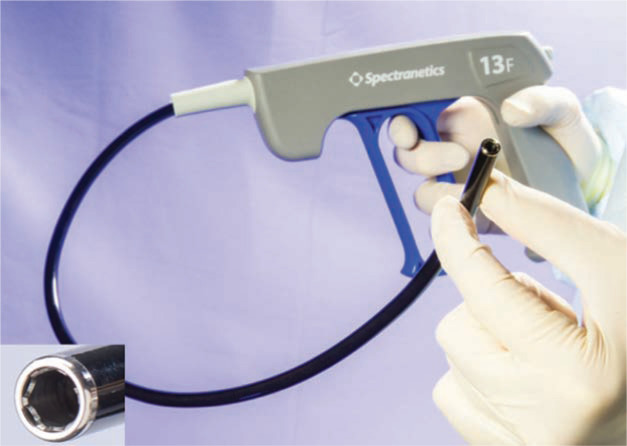
The TightRail™ Mechanical Dilator Sheaths (Spectranetics, Colorado Springs, CO, USA). The flexible shaft, shielded bidirectional rotating blade and static sheath are unique features that allow for the device to pass through dense and calcific scarring while requiring less traction force. The inset image is a magnified view of the sheath tip with the shielded metal blade. Courtesy of the The Spectranetics Corporation.

Despite the improved success rates of lead extraction with powered sheath technologies, the disruption of calcified binding sites remains difficult with either system. The most recent additions to the armamentarium of lead extraction tools provide a solution for this. The TightRail™ (9F, 11F, 13F) and TightRail Mini™ (9F, 11F) Mechanical Dilator Sheaths (Spectranetics, Colorado Springs, CO, USA) are “hand-powered” mechanical sheaths with a flexible shaft with a shielded metal dilating blade at the distal tip **(Figure 9)**. When activated, the blade extends 0.5 mm beyond the sheath with a bidirectional rotating mechanism that rotates a total of 574 degrees (287 degrees clockwise and 287 degrees counterclockwise). The outer aspect of the device is static and does not rotate with the dilating blade, making an outer sheath optional. The properties of a device with a flexible sheath, shielded blade, and static shaft offer significant advantages. The flexible nature of the shaft allows for the sheath to track the lead with less traction forces, reducing the need for significant traction forces to be present when navigating a high-risk structure like the superior vena cava with a laser sheath. The minimal traction forces required are also an advantage for leads with a higher risk of disruption because of the stress of excessive traction. The rotating blades are capable of cutting through dense and calcific scar tissue. Pressure must be applied on the shaft as close to the venous entry site as possible so that the distal end of the sheath can engage the binding sites. The shielded blade, which rotates in both directions with each activation, along with the non-rotating outer shaft, prevents “winding” of non-targeted leads. Despite all these technical advantages, however, sheath progression past binding sites is significantly lower than with use of the laser, and much more patience is required. Our personal experience with the TightRail™ Mechanical Dilator Sheath (Spectranetics, Colorado Springs, CO, USA) has led us to choose this tool as a first option for older leads.

**Figure 10:**
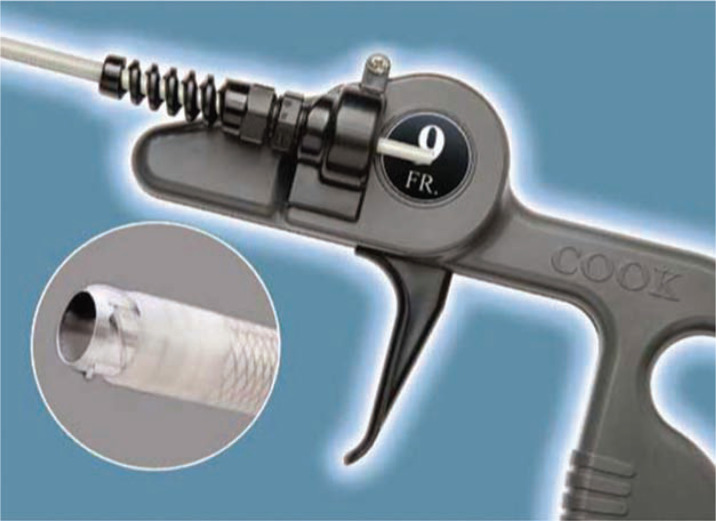
The Evolution^®^ and Evolution^®^ Shortie Mechanical Dilator Sheaths (Cook Medical, Bloomington, IN, USA). These “hand-powered” mechanical sheaths consist of a flexible, braided stainless steel sheath with a stainless steel spiral-cut dissection tip (inset). The sheath is attached to a trigger handle that rotates the sheath and allows for the threaded metal end to auger out the scar tissue. Courtesy of Cook Medical, Inc.

The Evolution^®^ and Evolution^®^ Shortie Mechanical Dilator Sheaths (Cook Medical, Bloomington, IN, USA) are other types of “hand-powered” mechanical sheath; each consists of a flexible, braided stainless steel sheath with a stainless steel spiral-cut dissection tip, and an outer dilating sheath. This sheath is attached to a trigger-activation handle that rotates the sheath and allows for the threaded metal end to bore through calcified and dense adhesions **(Figure 10)**.^32^ The Evolution^®^ sheath is quite useful for disrupting sites of calcified fibrosis, though this is often done at the expense of functional leads that were not intended targets for removal. Regardless, “hand-powered” mechanical cutting technology has provided an effective alternative for dealing with the challenges of densely scarred venous entry sites and heavily calcified adhesions.^33,34^

#### Femoral tools

**Figure 11:**
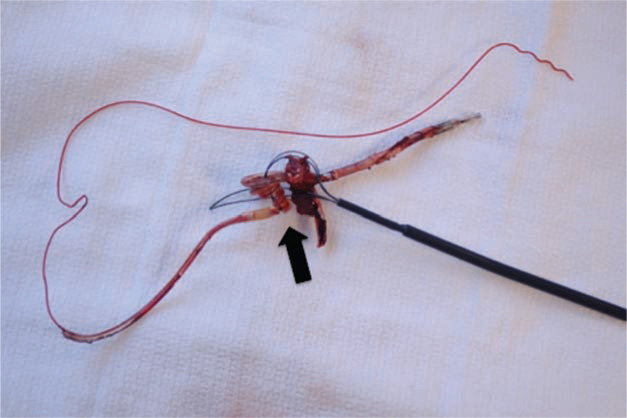
Transfemoral snaring of a lead. Transfemoral lead retrieval with the Byrd Workstation™ (Cook Medical, Bloomington, IN, USA) is a necessary skill for successful lead extraction, particularly in cases in which the lead is not accessible from the implant vein, as with a cut or fractured lead. Here, the lead has been snared and wound up by the Needle’s Eye^®^ snare (Cook Medical, Bloomington, IN, USA) (arrow), allowing for successful removal of the lead.

Transfemoral lead retrieval with the Byrd Workstation™ (Cook Medical, Bloomington, IN, USA) is a necessary skill for successful lead extraction. This is true particularly in cases in which the lead is not accessible from the implant vein, as in the case of a cut or fractured lead **(Figure 11)**. The Byrd Workstation™ (Cook Medical Medical, Bloomington, IN, USA) consists of a 16F outer sheath with a one-way valve that is advanced over a wire into the femoral vein, and a 12F inner sheath through which a number of retrieval snares can be advanced. The Byrd Workstation™ (Cook Medical) package contains a Needle’s Eye Snare^®^ (although other snare types can also be utilized, including the EN Snare^®^ Endovascular Snare System, Merit Medical Systems, Inc., South Jordan, UT, USA; and the Amplatz GooseNeck^®^ Snare, Covidien, Dublin, Ireland). If lead retrieval with a Needle’s Eye Snare^®^ (Cook Medical, Bloomington, IN, USA) proves unsuccessful, we have found that working with the combination of the Amplatz GooseNeck^®^ snare (Covidien, Dublin, Ireland) and bioptome forceps can also lead to success. In this situation, we preload the Amplatz GooseNeck^®^ snare (Covidien, Dublin, Ireland) onto the bioptome, advance the two together to the lead fragment, grasp the free lead tail with the bioptome, and then advance the snare over the bioptome to ensnare the lead.

The challenge of femoral retrieval remains as the ability to adequately manipulate the tools and snare the lead in three dimensions, while using two-dimensional fluoroscopic imaging. The recent description of a novel technology to facilitate extraction and maintenance of vascular access proposed a hybrid superior and inferior approach. This approach—which involves femoral snaring of the lead as a means to stabilize the lead while countertraction and counterpressure are used to free the lead—reinforces the clinical importance of femoral retrieval.^35^

#### Rescue tools

**Figure 12:**
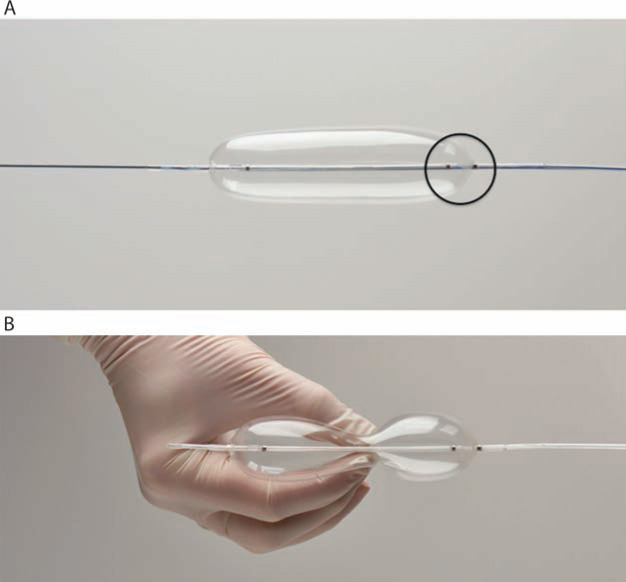
The Bridge™ Occlusion Balloon (Spectranetics, Colorado Springs, CO, USA). **A:** The Bridge™ balloon is an 8-cm, low-pressure compliant balloon designed to conform to the anatomy of the superior vena cava (SVC) in the majority of individuals. The proximal radiopaque marker (circled) should be positioned at the SVC-right atrial junction to insure complete coverage of the SVC. **B:** The Bridge™ balloon ((Spectranetics, Colorado Springs, CO, USA) is easily compressed with minimal pressure. Both images courtesy of The Spectranetics Corporation.

The performance of transvenous lead extraction has the potential for significant morbidity and mortality, and complications are inevitable. Although uncommon, vascular tears involving the superior vena cava (SVC) are often fatal. The single most important factor in preventing a major complication from leading to death is the time to intervention. To this end, a novel device has been developed to endovascularly tamponade the injury until definitive surgical repair can be performed. The Bridge™ Occlusion Balloon (Spectranetics, Colorado Springs, CO, USA) is a low pressure, compliant balloon measuring 8 cm in length that is designed to conform to the SVC in the majority of individuals **(Figure 12)**. The Bridge™ Occlusion Balloon (Spectranetics, Colorado Springs, CO, USA) can be deployed in under 2 min in the case of preexisting vascular access, and in animal models in less than 1 min when pre-positioned on a guidewire.^36^ Substantial real-world data are lacking, but in a porcine model, once deployed, the device reduces blood loss by 90% and affords approximately 30 min of acceptable hemostasis. Anecdotal reports and case series support the ability of the device to provide effective tamponade.^37^

### TLE techniques

If more than one lead is targeted for extraction, we often select the lead with the longer implant duration or the one that is suspected to become the most challenging to extract, to remove first. We have found that by selecting the initial lead to remove using these criteria, the scarring around the remaining lead(s) is sufficiently disrupted such that these lead(s) can be removed without the aid of an extraction sheath. When forward advancement of the sheath stalls, we pull the sheath back a short distance and rotate it slightly before re-engaging the scarred area. By approaching the binding area in a different plane, we are often able to successfully cross adhesions that initially posed challenges. If the impasse cannot be overcome with the aforementioned technique and the patient has another ipsilateral transvenous lead, we will switch our efforts to this other lead, sometimes sacrificing a lead that was not initially targeted for removal. In the case of a single transvenous lead, we would switch to another type of extraction sheath.

**Figure 13:**
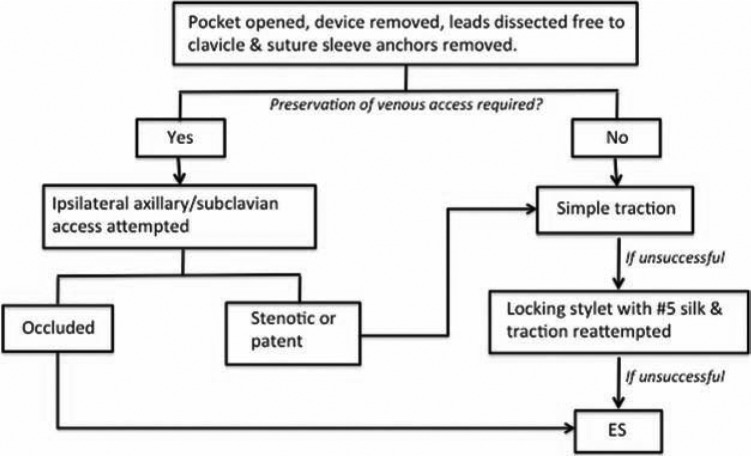
Stepwise approach to transvenous lead extraction. We routinely employ a stepwise approach to lead extraction so as to achieve the highest rate of complete success while utilizing the fewest number of tools. ES: extraction sheath.

In every case, we employ a stepwise approach to lead extraction with the goal of achieving complete success while utilizing the fewest tools **(Figure 13)**. Because we routinely perform TLE by super approach via the implant vein, the first step in the extraction procedure is making an appropriately positioned incision that permits easy access to the venous entry site in a plane parallel to the leads. It is our practice to attempt to use the existing incision whenever possible and perform an elliptical incision, excising the existing incisional scar. Occasionally, two incisions are necessary: one over the venous entry site of the leads, and a second over the pocket or area of skin erosion or adherence. Once the pocket is entered, microbial cultures of pocket tissue are obtained in all cases of CIED infection. Then, the device is removed and the leads are dissected free, back to their venous entry site. Dissection around the venous entry site is important because it allows for easy passage of any devices that may be necessary for lead extraction. However, aggressive dissection can result in transient problems with hemostasis secondary to back bleeding. Some operators opt to perform a loose figure-of-eight stitch around the venous entry site that can be easily pulled taut for quick hemostasis. The anchor sleeves are then removed, and all extraneous material, including suture material, is then eliminated from the pocket. Complete removal of infected tissue and foreign material is mandatory in cases of CIED infection. Additionally, we routinely perform a capsulectomy whenever ipsilateral reimplantation is planned.

If ipsilateral reimplantation is planned, ipsilateral venous access is attempted under fluoroscopic guidance with or without the aid of intravenous contrast. In our experience, stenotic lesions can often be crossed by using a 5F dilator and Terumo Glidewire^®^ (Terumo Interventional Systems, Somerset, NJ, USA). If the vein is successfully cannulated and a wire can be passed into the inferior vena cava (IVC), or if ipsilateral reimplantation is not planned, lead removal with simple traction is attempted. If this proves unsuccessful, then the lead is cut and a locking stylet with #5 silk is introduced, and traction is reattempted. The #5 silk is used to reinforce the lead and to prevent the insulation from bunching up or “snowplowing” under the force of counterpressure. Additionally, it has become our practice to routinely use one or more One-Tie^®^ device(s) on all implantable cardioverter-defibrillator leads and, specifically, leads that are at high-risk for disruption with traction (e.g. Fineline™; Boston Scientific, Marlborough, MA, USA). If lead removal still proves unsuccessful, a non-powered or powered sheath is employed. Sheath selection is determined by the clinical situation and the operator’s preference and experience. We have had excellent success utilizing a laser sheath as our tool of choice, but as mentioned earlier, we have learned to select a TightRail™ sheath (Spectranetics, Colorado Springs, CO, USA) for fragile or older leads. If the lead is not retrievable from the implant vein, or lead disruption occurs, then transfemoral retrieval is performed.

**Figure 14:**
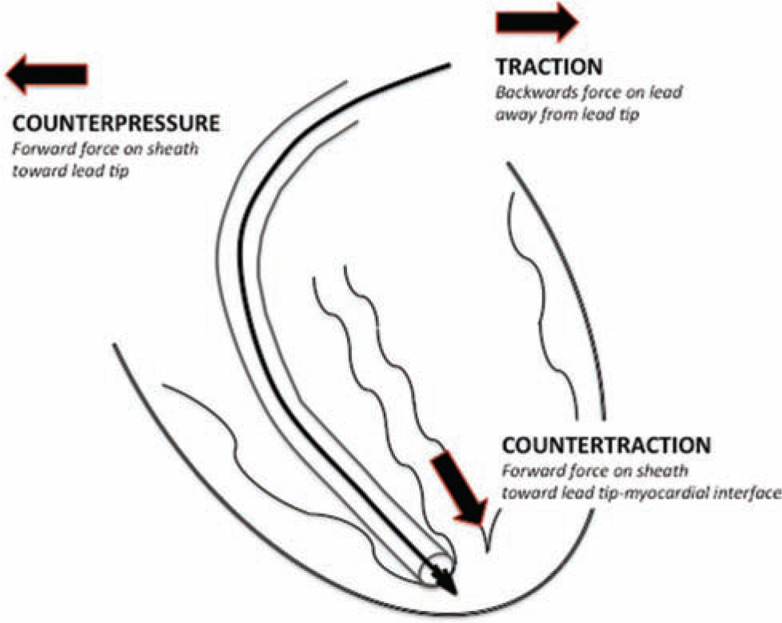
Schematic representation of the forces of counterpressure, traction and countertraction. Counterpressure is the force applied by the non-powered or powered sheath as it is advanced over the lead, interrupting areas of adherent scar tissue. Traction is the pulling force on the lead needed to provide a straight “rail” so as to allow for the sheath to follow the lead. Countertraction is the forward force applied by the sheath at the myocardium to limit the traction forces on an entrapped electrode to the circumference of the sheath at the lead tip-myocardium interface. Once the lead is released from the fibrous tissue, the myocardium falls away from the sheath, thereby reducing the risk of myocardial invagination or injury.

Counterpressure is the force applied by the non-powered or powered sheath as it is advanced over the lead, interrupting areas of adherent scar tissue. Sufficient traction must be applied to the lead and locking stylet so as to allow for the sheath to follow the lead body and not damage the vasculature as the lead curves within the vein. Countertraction is a technique employed once the sheath has been advanced to the lead tip-myocardium interface. Applying countertraction limits the traction forces on an entrapped electrode to the circumference of the sheath at the lead tip-myocardium interface. Once the lead is released from the fibrous tissue, the myocardium falls away from the sheath, thereby reducing the risk of myocardial invagination or injury **(Figure 14)**.^16,20,38^

### Lead extraction approach

Leads are typically extracted by a superior approach via the implant vein, although alternative approaches are used in certain situations. For example, when the free lead tip cannot be reached from the implant vein, an inferior approach via the femoral vein is necessary. Occasionally, hybrid or alternative venous approaches are utilized. Bongiorni and colleagues reported on the success of a combined approach via the femoral and internal jugular veins for free-floating leads and leads with dense SVC adhesions.^39^ Lead stabilization by femoral snaring via an inferior approach provides a straighter “rail” for an extraction approach from the right internal jugular vein, decreasing the likelihood of SVC avulsion. Recently, Fischer et al.^35^ described a hybrid superior and inferior approach with femoral snaring of the lead to provide stability from below while counterpressure and traction were applied from above. The researchers demonstrated this to be a safe and effective technique for lead extraction while maintaining venous access.

### Potential complications and management

Complications are inevitable, even among experienced extractionists **(Table 3)**. Rapidly recognizing and responding to complications are essential, and represent the most important factors in preventing a major complication from leading to death. Because different complications require different surgical approaches to treat or manage, rapid response requires not only the immediate availability of cardiac surgery, but also communication with the surgeon regarding the circumstances leading up to the complication and the believed anatomic location of the injury.

The most common causes of hypotension during TLE are vagal responses, cardiac tamponade and hemothorax. Immediately available echocardiography (transthoracic and transesophageal) is necessary for the prompt diagnosis and treatment of cardiac tamponade. In addition, all extractionists should know how and be able to perform emergent pericardiocentesis. In our experience, pericardial bleeding frequently stabilizes and stops following percutaneous drainage. However, surgery should be performed in all cases of continued significant pericardial bleeding. Bleeding into the pleural space can be transiently masked by aggressive fluid resuscitation and, thus, this needs to be considered in any patient requiring significant volume resuscitation for the maintenance of blood pressure. It is not uncommon for patients to lose several liters of blood into the pleural space before the problem is diagnosed. Vascular injuries above the pericardial reflection or to the SVC require immediate surgical intervention in almost all cases. Vagal responses occur commonly in the setting of direct myocardial traction and will respond to fluid resuscitation and atropine. The majority of vagal responses are self-limiting.

Less common complications of TLE include acute tricuspid valve injury, arteriovenous fistula, and venous thrombosis. Traumatic tricuspid regurgitation can frequently be managed medically, although surgical repair is sometimes necessary in severe cases. Arteriovenous fistulae can manifest acutely at the time of the procedure or several days postoperatively, and management ranges from observation to percutaneous or open surgical correction. Venous thrombosis probably occurs more commonly than is recognized, but anticoagulation is warranted only in symptomatic cases. In cases of pocket infection, pocket hematomas are common following debridement.

Despite technologic advances, TLE can be associated with significant morbidity and mortality. Reported major complication and mortality rates with transvenous lead extraction vary widely across studies, ranging from 0.4% to 7.3%.^27,39–43^ Complication rates with TLE directly parallel operator experience. Major and minor complications are reduced by approximately 50% with increased operator experience, from 20 to 120 cases, to >300 cases performed.^40^ Large-scale multicenter randomized trials have confirmed the effect of experience on outcomes.^27^ Likewise, observational registries of experienced, high-volume lead extractors have consistently demonstrated even higher success rates, with exceedingly low major complication and mortality rates.^39,42,43^ More recently, a European prospective registry of consecutive TLE procedures, the European Lead Extraction ConTRolled Registry (ELEC-TRa), observed that major complications and mortality with TLE were twice as high among high-volume centers, as compared with among low-volume centers.^44^

## Future directions

TLE has evolved dramatically over the past 30 years, and exponentially throughout the past decade. Despite these advances, however, the basic tenets of counterpressure, traction and countertraction remain critical to ensuring successful and safe outcomes. As indications for device therapy expand and younger patients receive devices, it is likely that the number of lead extractions performed will increase. As they do, the pressure on the field to continue to evolve lead extraction, not simply as a technique, but as a discipline and a science, will gain strength. The future of lead extraction lies in the development of new techniques, tools and training methods, the creation of a collaborative community, and the growth of the science.

## Figures and Tables

**Table 1:** Indications for Transvenous Lead Extraction^14^

	Infection	Thrombosis or Venous Stenosis	Functional Leads	Non-Functional Leads	Chronic Pain
**Class 1** Procedure *SHOULD* be performed	In cases of definite CIED infection (valvular endocarditis, DRE, sepsis) *(LOE: B)*. In cases of CIED pocket infection (abscess, erosion, chronic draining sinus *(LOE: B)*. In cases of valvular endocarditis w/o definite lead and/or device involvement *(LOE: B)*. With findings of occult gram-positive bacteremia *(LOE: B)*.	With clinically significant TE events associated w/ thrombus on lead or fragment *(LOE: C)*. With bilateral SCV or SVC occlusion precluding implant of needed TV lead *(LOE: C)*. With planned stent deployment in vein w/ TV lead already to avoid entrapment *(LOE: C)*. In cases of symptomatic SVC stenosis/occlusion *(LOE: C)*. In cases of ipsilateral venous occlusion precluding implant of an additional lead when a contralateral implant is contraindicated (AVF, shunt or vascular access port, mastectomy) *(LOE: C)*.	In cases involving lifethreatening arrhythmias due to retained leads *(LOE: B)*. When leads, due to design or failure, may pose immediate threat if left in place *(LOE: B)*. When leads may or do interfere w/ CIED function *(LOE: B)*. When leads are present that interfere w/ treatment of malignancy (radiation, surgery) (LOE: C).		
**Class IIa** It is *REASONABLE* to perform the procedure	With findings of persistent occult gram-negative bacteremia *(LOE: B)*.	With ipsilateral venous occlusion precluding ipsilateral implant of additional lead w/o contraindication of a contralateral implant *(LOE: C)*.		When leads, due to design or failure, are potentially dangerous if left in place *(LOE: C)*. When a CIED implant would yield >4 leads on one side or >5 leads through SVC *(LOE: C)*. When there is a need for MRI imaging w/o an alternative *(LOE: C)*.	When there is severe chronic pain at the device or lead insertion site w/ significant discomfort not manageable by medical or surgical techniques and when there is no acceptable alternative available *(LOE: C)*.
**Class IIab** Procedure *MAY BE CONSIDERED*			When there are leads present that may interfere w/ CIED function *(LOE: C)*. When leads, due to design or failure, are a potential threat if left in place *(LOE: C)*. When there are abandoned leads *(LOE: C)*. When there is a need for MRI imaging w/o an alternative available. *(LOE: C)*. When there is a need for an MRIconditional CIED system *(LOE: C)*.	At the time of the indicated CIED procedure w/o contraindication to TLE *(LOE: C)*. When there is a need for an MRI-conditional CIED system *(LOE: C)*.	
**Class III** Procedure should *NOT* be performed.	When there is superficial or incisional infection w/o involvement of device/ leads *(LOE: C)*. When there is chronic bacteremia due to a source other than CIED when long-term suppressive antibiotics are required *(LOE: C)*.		When there are redundant leads present with a <1 year life expectancy *(LOE: C)*. In cases involving known anomalous lead placement (SCA, Ao, pleura), or during placement through a systemic atrium or ventricle* *(LOE: C)*.	When there are redundant leads present with a <1 year life expectancy *(LOE: C)*. In cases involving known anomalous lead placement (SCA, Ao, pleura), or during placement through a systemic atrium or ventricle* *(LOE: C)*.	

*Can be considered w/ surgical backup. Adapted from the 2009 HRS Expert Consensus on Transvenous Lead Extraction.^14^ CIED: cardiovascular implantable electronic device; DRE: device-related endocarditis; TE: thromboembolic; SCV: subclavian vein; SVC: superior vena cava; SCA: subclavian artery; Ao: aorta; TV: transvenous; TLE: transvenous lead extraction; LOE: level of evidence; MRI: magnetic resonance imaging; w/: with; w/o: without.

**Table 2:** Transvenous Lead Extraction Cart Contents

• Standard stylets
• Wrenches
• Fixation tools
• Introducer sheaths
• Venous access kits
• Coronary sinus sheaths (with valves and splitters)
• Intravenous contrast
• Insulation repair kits
• IS-1 and DF-1 pins
• Locking stylets (LLD^®^, Spectranetics, Colorado Springs, CO, USA; and Liberator^®^, Cook Medical, Bloomington, IN, USA)
• #5 silk
• Bulldog™ Lead Extenders (Cook Medical, Bloomington, IN, USA)
• One-Tie^®^ Compression Coils (Cook Medical, Bloomington, IN, USA)
• Mechanical dilating sheaths
• Evolution^®^ (11 Fr, 13 Fr) and Evolution^®^ Shortie (9 Fr, 11 Fr) sheaths (Cook Medical, Bloomington, IN, USA)
• TightRail™ (9 Fr, 11 Fr, 13 Fr) and TightRail Mini™ (9 Fr, 11 Fr) sheaths (Spectranetics, Colorado Springs, CO, USA)
• Laser sheaths (12 Fr, 14 Fr, 16 Fr) and outer sheaths (S, M, L)
• Byrd Workstation™ (Cook Medical, Bloomington, IN, USA)
• Extraction snares (Needle's Eye^®^, Cook Medical, Bloomington, IN, USA; EN Snare^®^, Merit Medical Systems, Inc., South Jordan, UT, USA; and Amplatz GooseNeck^®^, Covidien, Dublin, Ireland)
• Bridge™ Occlusion Balloon (Spectranetics, Colorado Springs, CO, USA)
• Bioptome forceps
• Blazer radiofrequency ablation catheter (medium and large curve)
• Temporary pacing equipment
• Pericardiocenteis tray
• Vacuum containers for pericardial/chest tube drainage

**Table 3:** Potential Complications of TLE

Minor Complications	Major Complications
• Pericardial effusion not	• Death
requiring Intervention	• Cardiac avulsion
• Hemothorax not requiring	requiring intervention
intervention	(either percutaneous
• Pocket hematoma	or surgical)
requiring reoperation	• Vascular injury
• Upper extremity	requiring intervention
thrombosis resulting in the	(either percutaneous
need for medical	or surgical)
treatment	• Pulmonary embolism
• Vascular repair near	requiring surgical
implant or venous entry	intervention
sites	• Respiratory arrest/
• Hemodynamically	anesthesia-related
significant air embolism	complication prolonging
• Migrated lead fragment	hospitalization
without sequelae	• Stroke
• Blood transfusion as a	• CIED infection at
result of intraoperative	previously non-infected
blood loss	site
• Pneumothorax requiring	
a chest tube	
• Pulmonary embolism	
not requiring surgical	
intervention	
